# Plant Growth-Promoting Microorganism *Pseudarthrobacter* sp. NIBRBAC000502770 Enhances the Growth and Flavonoid Content of *Geum aleppicum*

**DOI:** 10.3390/microorganisms10061241

**Published:** 2022-06-17

**Authors:** Seung Hee Ham, A Ra Yoon, Hyun Eui Oh, Yoo Gyeong Park

**Affiliations:** 1 National Institute of Biological Resources (NIBR), 1008-11, Sangnam-ro, Sangnam-myeon, Miryang 50452, Korea; gka7278@korea.kr (S.H.H.); yoonara@korea.kr (A.R.Y.); hy1906@korea.kr (H.E.O.); 2 Department of Bio Health Science, Changwon National University, Changwon 51140, Korea; 3 Department of Horticultural Science, Chungnam National University, Daejeon 34134, Korea

**Keywords:** indole-3-acetic acid, native plant, plant growth-promoting rhizobacteria (PGPR)

## Abstract

Plant growth-promoting rhizobacteria are known to enhance the growth and antioxidant activity of several plants. However, the effects of such rhizobacteria on *Geum aleppicum*, a plant with pharmacological potential in Korea are unknown. In this study, we investigated the effects of *Pseudarthrobacter* sp. NIBRBAC000502770 treatment (100 mL/pot, every two weeks for 55 days), in the form of culture medium, 100−fold diluted culture, culture supernatant, and pelleted cells resuspended in water, on the growth, antibacterial activity and flavonoid content of *G. aleppicum*. The NIBRBAC000502770 strain showed high indole-3-acetic acid (IAA) content of 461.81 μg∙mL^−1^. The dry weight of the roots was significantly higher in the supernatant, diluted culture, and pellet-treated plants compared to that in the control plants. Additionally, the plant height, root length, leaf length, leaf width, chlorophyll content, biomass, and dry weight of the shoot were highest in the pellet-treated plants. Further, methanol extracts of pellet-treated plants showed significantly high flavonoid content compared to that in the control plants (28 mg∙g^−1^ vs. 7.5 mg∙g^−1^) and exhibited strong antibacterial activity against Gram-positive and negative bacteria. These results demonstrate the beneficial effects of *Pseudarthrobacter* sp. NIBRBAC000502770 on the growth and flavonoid content of *G. aleppicum.*

## 1. Introduction

The genus *Geum* belongs to the Rosaceae family and is widely distributed in temperate regions [1]. There are about 40 species distributed worldwide, including wild and cultivated perennial herbaceous plants [2]. Since 1920, more than 200 compounds have been isolated from the genus *Geum*, including monoterpenoids, sesquiterpenes, triterpenoids, flavonoids, hydrolysable tannins, and phenylpropanoids [3]. Many *Geum* species have pharmacological potential due to the abundance of these biologically active compounds [4].

Among the *Geum* species, *Geum* *aleppicum* grows in valleys and is distributed in Japan, Mongolia, Siberia, and Europe [5,6]. In particular, *G. aleppicum*, in China, contains benzoic acid, gallate acid, salicylic acid, vanillin, 3,4,5-trihydoxybenzoic dehyde, and 3,4,5-trihydroxybenzoic acid ethyl ester [7], and has been used as a diuretic and astringent [3]. Despite the high potential for medicinal use of *G. aleppicum*, which grows wild in Korea, related studies are insufficient. According to a study, the extract of *G. aleppicum*, which is native to Korea, exhibited free radical scavenging activity, protective effect against cell damage caused by free radicals, tyrosinase inhibitory activity, and reduction of elastase activity, and has the potential to be used as a raw material for functional cosmetics [5].

Recently, research on antibacterial effects among the various physiologically active effects of Korean native plants has been attracting increasing interest in food, cosmetics, livestock, and other related fields. The physiologically active substances of Korean native plants are known to have antioxidant, antibacterial, and anti-inflammatory functions, and are widely used as raw materials for pharmaceuticals today. In addition, the development of antibacterial agents using these physiologically active substances is required due to the safety and resistance issues of synthetic agents. Therefore, studies on the isolation of antibacterial substances from various Korean native plant and confirmation of their antibacterial activity are being actively conducted. The antibacterial activity of *Geum urbanum* L. methanol extract was shown in *Bacillus cereus* and *Staphylococcus aureus* [4]. Similarly, the antibacterial activity of *Geum japonicum* Thunb. methanol extract was shown in *Staphylococcus epidermidis* and *Pseudomonas aeruginosa* [8]. In addition, *Geum rivale* methanol extract showed higher antibacterial activity in Gram-positive bacteria than in Gram-negative bacteria [9].

Among various methods, treating plants with plant growth-promoting rhizobacteria (PGPR) was shown to increase their antioxidant activity. PGPR are soil microorganisms in the rhizosphere, that have a beneficial effect on plant growth and productivity. They are involved in important life processes such as plant growth and development and productivity by influencing plant hormone regulation and nutrient absorption and utilization [10]. PGPR and plants exhibit a mutual symbiotic relationship in which, plants provide PGPR with food and PGPR exert beneficial effects on plant growth by providing plants with nutrients and fixing nitrogen [11]. For example, four species of *Bacillus* genus increased the growth of soybean and wheat [12], and *pseudomonas fluorescens* improved the growth and biomass yield of *Curcuma longa* L. (turmeric) [13].

Due to the growing interest in sustainable eco-friendly organic agriculture around the world, the importance of bio-fertilizers is rising, and various useful microorganisms are being reported by many researchers. Although many studies have been conducted to investigate the effects of PGPR on crops, studies on the interaction and effects of PGPR on Korean native plants are lacking. Moreover, to the best of our knowledge, there have been no studies on the growth and antioxidant activity of *G. aleppicum* treated with PGPR. Therefore, this study will be one of the important the first to demonstrate the effect of PGPR on the promotion of growth and increase of phenol and flavonoid content in Korean native plants. Mass production and use of these useful microorganisms as biofertilizers may contribute greatly to the growth of native plants.

In this study, we demonstrated the effects of plant PGPR *Pseudarthrobacter* sp. NIBRBAC000502770 isolated from soil, on the growth, antibacterial activity, and phenol and flavonoid content in Korean native *G. aleppicum* plants.

## 2. Materials and Methods

### 2.1. Bacterial Strains

Auxin-producing strain, *Pseudarthrobacter* sp. NIBRBAC000502770 was isolated from the soil of a shooting range in Hongcheon-gun, Gangwon-do, Korea [14]. The strain was inoculated in LB broth and cultured at 30 °C and 180 rpm for 48 h, followed by centrifugation at 4 °C, 14,000× *g* and 10 min to recover the supernatant. To confirm the auxin-producing ability, the supernatant and Salkowski reagent were mixed in a ratio of 1:2 and allowed to react in the dark for 30 min, and the absorbance was measured at 530 nm using a spectrophotometer [15]. It was quantified using a standard curve obtained using standard Indole-3-acetic acid (IAA) (Sigma Chemical Co., St. Louis, MO, USA). In order to examine the growth promoting effect of the strain producing 461.8 μg·mL^−1^ of IAA, a control group and four experimental groups were used. In the “culture solution” group, the *Pseudarthrobacter* sp. NIBRBAC000502770 strain was cultured in LB broth and the culture was used as such. In the “diluted solution” group, a 100−fold diluted culture solution was used. In the “supernatant” group, the supernatant obtained by centrifuging the culture solution (14,000× *g*, 15 min) was used. In the “pellet” group, cells were recovered by centrifugation and cell suspension (1 × 10^7^ cells·mL^−1^) diluted with distilled water was used. Treatment was carried out by inoculating the above suspensions in each experimental group, into the rhizospheres of the *G. aleppicum* (100 mL per pot at intervals of 2 weeks). The control group was treated only with water.

### 2.2. Plant Materials and Growth Conditions

*G*. *aleppicum* seeds (NIBRGR0000188177) were provided by the National Institute of Biological Resources. Seedlings at 6 weeks after sowing (average plant length 6.18 cm, average number of leaves 6.80, average root length 5.88 cm, average fresh weight 0.54 g) were used for experimentation. The seedlings were transplanted into a pot with a diameter of 10 cm and grown in a greenhouse for 55 days from 21 July to 13 September 2021. During the experiment, the average temperature of the greenhouse was 35.3 °C and the average relative humidity was 57.9%, and the pots were irrigated with tap water three times a week.

### 2.3. Data Collection and Statistical Analysis

#### 2.3.1. Growth Characteristics

The number of *G. aleppicum* per experimental group was performed in three replications of three each. To analyze the growth of *G*. *aleppicum*, the following growth parameters were measured after 55 days of treatment: plant height, leaf length, leaf width, leaf number, chlorophyll, stem diameter, fresh weight, dry weight, and root length. Leaf length and leaf width were measured for the largest leaf, and root length was measured for the longest root. Chlorophyll was measured using a chlorophyll meter (SPAD-502 Plus; Konica Minolta Sensing Inc., Osaka, Japan), and stem diameter was measured using a digital caliper (CD-20; Mitutoyo Corp., Kanagawa, Japan). Fresh and dry weights were measured using microbalance (ME204; Mettler-Toledo, Greifensee, Switzerland). Dry weights were measured after drying the plants at 60 °C for 48 h using a dry oven (SJ202-DM; Sejong Scientific Co., Ltd., Bucheon, Korea).

#### 2.3.2. Extract Preparation

After 5 weeks of growth, *G*. *aleppicum* shoots were harvested, pulverized using liquid nitrogen, and used for antioxidant analysis. The pulverized sample (100 mg) was incubated with 1 mL of 50% methanol for 6 h. Then, the reaction product was centrifuged at 24,000× *g* for 20 min at 4 °C to obtain the methanol extract (5424R, Eppendorf, Hamburg, Germany).

#### 2.3.3. Antibacterial Activity

A total of nine bacterial strains (six Gram-positive and three Gram-negative) were used in this experiment. The bacterial strains were inoculated into 10 mL of Nutrient broth (Difco Co., Sparks, MD, USA), LB broth (Difco, USA), and BHI broth (Difco, USA), and cultured for 24–48 h at 37 °C. The antibacterial activity of the extracts of *G*. *aleppicum* was analyzed using an agar-well diffusion assay [16]. Each bacterial culture was adjusted to an optical density (O.D) value of 0.2 at 600 nm, and the test agar plates were prepared by inoculating with 1% culture medium. A well was made in the test plate and 50 μL of *G*. *aleppicum* extract was loaded and left on a clean bench for 30 min, allowing the extract to spread. The plates were then incubated for 24 h at 37 °C. Antibacterial activity was confirmed by measuring the size (mm) of the clear zone around the well where the growth of bacteria was inhibited.

#### 2.3.4. Total Phenol Content

The total phenol content in *G. aleppicum* extract was measured by the method described by Thimmaiah [17]. Briefly, 450 μL of distilled water was added to 50 μL of the extract and 500 μL of 50% Folin–Ciocalteu reagent and 500 μL of 2.5% sodium carbonate solution was added and incubated for 40 min in the dark. After the reaction, absorbance was measured at 765 nm using a spectrophotometer (UV-1280, Shimadzu, Japan). The standard curve of total phenol was prepared using quercetin.

#### 2.3.5. Total Flavonoid Content

The total flavonoid content of *G*. *aleppicum* extract was measured according to the method described by Quettier-Deleu [18]. Briefly, 50 μL of the extract was added to a mixture of 450 μL 80% methanol and 500 μL 2% AlCl_3_ and incubated at room temperature for 30 min, following which the absorbance was measured at 415 nm using a spectrophotometer (UV-1280, Shimadzu, Japan). The total flavonoid content was calculated using quercetin as the standard.

#### 2.3.6. Statistical Analysis

Statistical analysis was performed using SAS version 9.4 (Cary, NC, USA). Data were compared using analysis of variance (ANOVA) and Duncan’s multiple range tests and *p* ≤ 0.05 was considered statistically significant.

## 3. Results

### 3.1. Effects of Pseudarthrobacter sp. Treatment on Growth Characteristics of G. aleppicum

**Figure 1:**
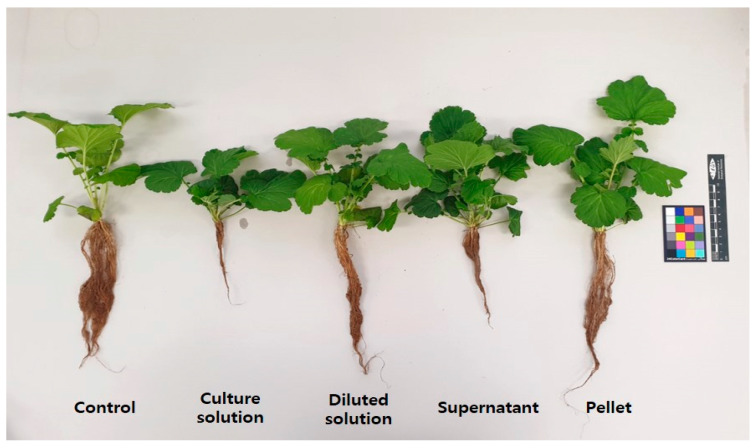
Growth and development of *Geum aleppicum* after 55 days of treatment with *Pseudarthrobacter* sp. NIBRBAC000502770. Control: tap water; Culture solution: the *Pseudarthrobacter* sp. NIBRBAC000502770 strain was cultured in LB broth; Diluted solution: a 100−fold diluted culture solution; Supernatant: obtained by centrifuging the culture solution (14,000× *g*, 15 min); Pellet: cells were recovered by centrifugation and cell suspension (1 × 10^7^ cell∙mL^−1^) diluted with distilled water was used.

**Figure 2:**
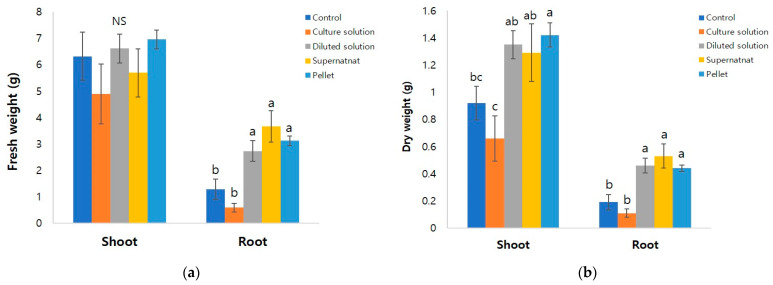
Effect of different treatments for 55 days on fresh weight (**a**) and dry weight (**b**) of shoot and root of *Geum aleppicum*. Control: tap water; Culture solution: the *Pseudarthrobacter* sp. NIBRBAC000502770 strains was cultured in LB broth; Diluted solution: a 100−fold diluted culture solution; Supernatant: obtained by centrifuging the culture solution (14,000× *g*, 15 min); Pellet, cells were recovered by centrifugation and cell suspension (1 × 10^7^ cell·mL^−1^) diluted with distilled water was used. Vertical bars indicate standard error of the means. Data are represented as mean ± S.E of the 3 biological replicates. Different letter (a,b,c) on bar graphs indicate the significant differences between treatment conditions by using Duncan’s multiple range test at *p* < 0.05. NS: non-significant.

The treatment and treatment type of *Pseudarthrobacter* sp. had a significant effect on the growth of *G*. *aleppicum* at the end of the experiment (Figure 1). After 55 days of treatment, the average plant height was the highest at 19.47 cm when *Pseudarthrobacter* sp. was treated in pellet form, and the lowest at 12.64 cm when treated with the culture solution (Table 1). The pellet treatment also had the average leaf length and width of 7.26 cm and 8.73 cm, respectively, but there was no significant difference among the treatment groups (Table 1). The number of leaves was the most at 81.1 in the supernatant group, and the lowest at 61.0 in the culture solution group, but there was no significant difference among the treatment groups (Table 1). The chlorophyll content was also highest in the pellet treated group, but was not significantly different among the treatment groups. The stem diameter was the highest at 2.79 mm in the pellet treated group, and the lowest at 1.91 mm in the supernatant treated group (Table 1). The fresh weight of the shoot was the highest at 6.95 g in the pellet treated group, but it was not significantly different among the treatment groups (Figure 2A). The dry weight of the shoot was also the highest at 3.12 g in the pellet treated group, which was about 1.5 times higher than that of the control group (Figure 2B). The fresh weight of the root was the highest at 3.67 g in the supernatant treated group, and the pellet treated group was the second highest at 3.12 g (Figure 2A). The dry weight of the root was highest at 0.53 g in the supernatant treated group, followed by 0.46 g in the diluted culture solution treated group, and 0.44 g in the pellet treated group (Figure 2B). These values were 2.8, 2.4, and 2.3 times higher than that of the control group, respectively. The root length was the longest at 29.93 cm in the pellet treatment group, and the shortest at 13.71 cm in the culture solution treated group (Table 1, Figure 2).

### 3.2. Antibacterial Activity

The antibacterial activity of the *G. aleppicum* against nine bacterial species was confirmed using the methanol extracts of shoots of the plants from each treatment group. Among the Gram-positive bacteria, the extracts showed highest antibacterial activity against *Micrococcus luteus*, which inhibition zones of 13 mm, 15 mm, and 16 mm diameter in culture solution, supernatant, and pellet-treated groups respectively (Table 2). The second highest activity among Gram-positive bacteria was against *Bacillus cereus*, which showed inhibition zones of 9 mm, 10 mm, and 11 mm in culture solution, supernatant and pellet treated groups respectively (Table 2). Among Gram-negative bacteria, *Salmonella enteritidis* and *Shigella boydii* showed 8 mm inhibition zones in culture solution treated group and 10 mm inhibition zones in supernatant and pellet treated groups (Table 2). In addition, in *Escherichia* *coli*, extracts from the supernatant and pellet treated groups showed inhibition zones of 9 mm (Table 2).

### 3.3. Total Phenol and Flavonoid Contents

**Figure 3:**
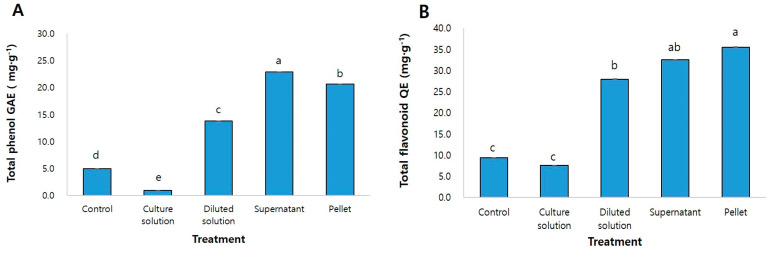
Total phenol (**A**) and total flavonoid (**B**) of methanol extracts of *Geum aleppicum* affected by strain treatments. Control: tap water; Culture solution: the *Pseudarthrobacter* sp. NIBRBAC000502770 strains was cultured in LB broth; Diluted solution: a 100−fold diluted culture solution; Supernatant: obtained by centrifuging the culture solution (14,000× *g*, 15 min); Pellet, cells were recovered by centrifugation and cell suspension (1 × 10^7^ cell·mL^−1^) diluted with distilled water was used. Vertical bars indicate standard error of the means. Data are the mean ± S.E of the 3 biological replicates. Different letter (a,b,c,d,e) on bar graphs indicate the significant differences between treatment conditions by using Duncan’s multiple range test at *p* < 0.05.

The phenol and flavonoid contents present in the methanol extract of *G. aleppicum* were measured using quercetin as a standard. The total phenol content was as high as 22.9 mg·g^−1^ in the supernatant treated group, and 20.6 mg·g^−1^ in the pellet treated group (Figure 3A). In addition, the total flavonoid content was 35.5 mg·g^−1^ in the pellet, which was 3.8 times higher than that of the control, followed by 32.5 mg·g^−1^ of the supernatant, which was 3.5 times higher than that of the control (Figure 3B).

## 4. Discussion

The results show that treatment with plant growth-promoting rhizobacteria (PGPR) in various forms produced significant and different effects on plant growth. It is known that the growth and development of many plant species improved after inoculation with PGPR [19,20]. Some of the direct or indirect mechanisms that promote plant growth and development by PGPR inoculation are: (1) production of plant hormones such as indole-3-acetic acid; (2) improved plant tolerance to several environmental stress factors; and (3) suppression of soil-borne pathogens [21]. Plants respond to all plant hormones supplied from outside or produced by microorganisms that inhabit the rhizosphere. Soil microorganisms, especially the rhizosphere bacteria, can directly affect plants by producing plant hormones such as auxin, gibberellin, cytokinin, and ethylene [22,23]. Among them, IAA initiates roots and influences cell division and expansion, thereby increasing root surface area and consequently positively affecting the access to soil nutrients [20,24]. Plants treated with IAA for a long period of time develop roots, and absorb more nutrients, contributing to the growth of the plant [25]. In our study, after inoculating with *Pseudarthrobacter* sp. NIBRBAC000502770, a strain that produces 461.8 μg·mL^−1^ of IAA, the growth *G. aleppicum*, were significantly enhanced. The fresh and dry weight of the root were increased by more than two to three times compared to the control (Figure 2), which indicates the stimulation of root development by IAA producing *Pseudarthrobacter* sp. NIBRBAC000502770.

It is known that PGPR promote plant growth through the production of plant hormones, and by synthesizing bioactive compounds and activating the plant defense system as well. Several studies have reported that beneficial microbes associated with roots play an important role in increasing the growth and the phytochemical content of medicinal plants which are used to treat various diseases [26,27]. Phenol is a secondary metabolite related to plant color, quality, nutrition, and antioxidant properties. Phenolic compounds have one or more aromatic rings with one or more hydroxyl groups, and can be classified into flavonoids and non-flavonoids. Flavonoids are based on a flavonoid nucleus consisting of three phenolic rings called A, B, and C [28]. It is known that the number and position of hydroxyl groups on phenolic groups is related to the relative toxicity against microorganisms, and that increased hydroxylation increases toxicity [29]. Flavonoids inhibit bacterial growth by various mechanisms, such as inhibiting the synthesis of nucleic acids having B-ring hydroxylation [30] or inhibiting cytoplasmic membrane function [31]. As such, flavonoids protect plants from various biological and abiotic stresses and play an important role in the interaction between plants and their environment. Therefore, in this study, the antibacterial activity of *G. aleppicum* methanol extract and changes in total phenol and flavonoid contents were confirmed to investigate the relationship between PGPR and the physiologically significant compounds in the plant.

PGPR inoculation is known to produce effective antibacterial compounds against soil pathogens and pests [32,33], and our data confirmed that antibacterial activity of the plant extract significantly increased when PGPR was inoculated in the form of pellet. Extracts from plants treated with the culture supernatant and pellet of *Pseudarthrobacter* sp. showed the highest antibacterial activity against *Micrococcus luteus*. In addition, antibacterial activity was also observed against *Bacillus cereus*, *Escherichia coli*, *Listeria monocytogenes*, *Salmonella enteritidis*, *Shigella boydii*, *Staphylococcus aureus,* and *Streptococcus mutans*. Similar results in previous studies show that the plant extracts exhibit higher activity against Gram-positive bacteria than Gram-negative bacteria [34,35]. The reason for the difference in activity against Gram-positive and Gram-negative bacteria is thought to be due to the difference in the composition of the bacterial biological membrane. Although the cell wall of Gram-negative bacteria is thinner than that of Gram-positive bacteria, the structure is more complex, and it is surrounded by an outer membrane, preventing the absorption of substances from the outside. In addition, the surface of the outer membrane is known to play an important role in avoiding phagocytosis and complement action, and it is known to increase resistance to many toxic substances by acting as a barrier for material permeation [36]. Because of these roles, the outer membrane acts as a barrier to the permeation of peptides, causing their activity to be lower in Gram-negative bacteria than that in Gram-positive bacteria. However, the antibacterial activity of some Gram-negative bacteria is also classified into Gram-negative and positive bacteria according to the bacterial classification, but since there is a difference in each cell structure, it is judged that this is due to the difference in characteristics between each strain. Previous studies have shown the antibacterial activity of various *Geum* species, including the activity of *G. aleppicum* against Gram-positive bacteria.

Our results showed that the total phenol and flavonoid content were increased when *G. aleppicum* was treated with PGPR in the form of a diluted solution or pellet. In a similar study, inoculation of *Origanum* L. with *Pseudomonas* sp. increased its phenolic content [37], and treatment of tomatoes with *B. licheniformis* increased the total flavonoid content [38]. PGPR is known to regulate the level of plant metabolites such as phenols, sugar, amino acids, and organic acids by increasing the activity of enzyme involved in their biosynthesis [39]. In particular, plants treated with PGPR were shown to have increased expression of peroxidase and polyphenol oxidase enzymes involved in the phenol and flavonoid metabolism [40,41]. As a result, treatment of *G. aleppicum* with promotes plant growth by increasing secondary metabolites such as phenol and flavonoids.

## 5. Conclusions

In conclusion, our results show that *Pseudarthrobacter* sp. NIBRBAC000502770 strain has high IAA content of 460 μg·mL^−1^. Treatment of *G. aleppicum* plants with this strain of PGPR enhanced the growth of the shoot and the root. In addition, our results showed the antibacterial activity and high phenol and flavonoid content in the methanol extracts of *G. aleppicum* plants treated with the strain. Therefore, the positive effects of *Pseudarthrobacter* sp. NIBRBAC000502770 in promoting the growth, antibacterial and antioxidant activity of *G. aleppicum* make it an eco-friendly biofertilizer with potential applications in small household farms and large-scale agriculture.

## Figures and Tables

**Table 1:** Effect of *Pseudarthrobacter* sp. treatment on the growth and development of *Geum aleppicum* plants.

Treatment ^z^	Plant Height (cm)	Leaf Length (cm)	Leaf Width (cm)	No. of Leaves	Chlorophyll (SPAD)	Stem Diameter (mm)	Root Length (cm)
Control	17.19 ^ay^	6.31	7.44	67.22	32.26	2.36 ^ab^	27.20 ^a^
Culture solution	12.64 ^b^	5.88	6.94	61.00	33.03	1.96 ^a^	13.71 ^b^
Diluted solution	17.37 ^a^	6.68	8.04	70.22	31.78	2.64 ^a^	28.20 ^a^
Supernatnat	13.40 ^b^	5.60	6.89	81.11	33.99	1.91 ^b^	14.54 ^b^
Pellet	19.47 ^a^	7.26	8.73	69.78	34.98	2.79 ^a^	29.93 ^a^
F-test	**	NS	NS	NS	NS	**	***

^z^ Control: tap water; Culture solution: the *Pseudarthrobacter* sp. NIBRBAC000502770 strains was cultured in LB broth; Diluted solution: a 100−fold diluted culture solution; Supernatant: obtained by centrifuging the culture solution (14,000× *g*, 15 min); Pellet: cells were recovered by centrifugation and cell suspension (1 × 10^7^ cell·mL^−1^) diluted with distilled water was used. ^y^ Different letter (a,b) on bar graphs indicate the significant differences between treatment conditions by using Duncan’s multiple range test at *p* < 0.05. NS, **, ***: non-significant or significant at *p* ≤ 0.01 or 0.001, respectively.

**Table 2:** Antibacterial activity of *Geum aleppicum* methanol extracts on various microorganisms on in agar well diffusion assay.

	Inhibition Zone of Plate (mm) ^1^				
Test Strains	Control	Culture Solution	Diluted Solution	Supernatant	Pellet
Gram-positive bacteria					
*Bacillu* *hs cereus*	- ^2^	9	-	10	11
*Staphylococcus aureus*	-	-	-	8.5	7.5
*Micrococcus luteus*	-	13	-	15	16
*Listeria monocytogenes*	-	-	-	9	8
*Enterococcus faecalis*	-	-	-	-	-
*Streptococcus mutans*	-	-	-	9.5	7.5
Gram-negative bacteria					
*Salmonella enteritidis*	-	8	-	10	10
*Escherichia coli*	-	-	-	9	9
*Shigella boydii*	-	8	-	10	10

^1^ Diameter (mm), ^2^ No antibacterial activity was observed.

## Data Availability

Data is contained within the article.
